# Dynamic Quality Prediction and Intelligent Classification of *Litopenaeus vannamei* in Cold Chain Based on Tad-Transformer

**DOI:** 10.3390/foods15152606

**Published:** 2026-07-25

**Authors:** Wei Dong, Min Niu, Huan Jiang, Liya Liu, Jiahui Zhang, Qingchuan Zhang

**Affiliations:** 1 School of Artificial Intelligence, Beijing Institute of Economics and Management, Beijing 100102, China; dongwei_06@126.com (W.D.); liyaliu@biem.edu.cn (L.L.); 17832908615@163.com (J.Z.); 2 National Engineering Research Centre for Agri-Product Quality Traceability, Beijing Technology and Business University, Beijing 100048, China; 18534068889@163.com

**Keywords:** *Litopenaeus vannamei*, cold chain quality, Tad-Transformer, time-series prediction, clustering algorithm

## Abstract

Temperature fluctuations in cold chain logistics accelerate protein degradation, lipid oxidation, and color deterioration of *Litopenaeus vannamei*, reducing product value and compromising food safety. To address this issue, this paper proposes a dynamic quality prediction method based on the Tad-Transformer neural network. First, storage experiments were conducted under six cold chain temperature conditions to collect multi-dimensional physicochemical and texture data. Core quality indicators were selected through temperature sensitivity analysis, and a time-series dataset was constructed. Second, an improved K-means++ clustering algorithm incorporating min-max constraints was applied for quality grading. Finally, the Tad-Transformer model was employed to predict quality indicators and temporal grade evolution. Comparative validation with Transformer, Informer and FEDformer on the self-constructed dataset demonstrates that, for the most challenging high-quality samples, the proposed model achieves both precision and recall exceeding 89%, representing improvements of 4.17–10.77% and 2.28–8.37%, respectively, over the comparison models. This method provides technical support for quality grading control and early warning of abnormal risks in cold chain logistics, offering a scientific reference for dynamic quality monitoring and intelligent evaluation of aquatic products.

## 1. Introduction

*Litopenaeus vannamei* is a globally significant aquaculture species with high economic value, characterized by high protein content, low fat levels, and a balanced amino acid profile [1]. As the global aquaculture industry continues to expand, farmed shrimp have progressively replaced wild-caught counterparts and now occupy a dominant position in international seafood trade [2,3]. However, post-harvest *L. vannamei* is highly susceptible to quality deterioration during cold chain logistics, which severely impedes the high-quality development of the shrimp industry. Factors such as temperature fluctuations, mechanical compression, and prolonged storage trigger endogenous enzymatic reactions and microbial proliferation, leading to melanosis, protein degradation, lipid oxidation, textural softening, and off-odor formation, thereby substantially reducing the commercial and edible value of the shrimp [4,5,6].

Quality deterioration in *L. vannamei* is primarily governed by polyphenol oxidase (PPO)-mediated enzymatic browning and microbial spoilage. Following harvest, metabolic disorders and disruption of cellular integrity facilitate sufficient contact between PPO and endogenous phenolic substrates, thereby initiating enzymatic browning reactions [7,8]. Mechanical stress during cold chain transportation further damages shrimp tissues and accelerates melanin deposition at the interface between the shell and muscle [9]. Temperature serves as a critical regulator of enzymatic activity and microbial growth. Elevated ambient temperatures significantly enhance PPO activity and accelerate the rate of melanosis, whereas imbalances in low-temperature conditions substantially promote the proliferation of dominant spoilage bacteria, including Pseudomonas, Shewanella, and Lactobacillus. This strong temperature-dependent deterioration mechanism renders cold chain temperature fluctuations a primary risk factor for rapid quality degradation of frozen and chilled shrimp.

To accurately assess shrimp freshness and spoilage levels, a comprehensive quality evaluation system encompassing sensory assessment, physical texture analysis, chemical assays, and microbiological detection has been established [10,11]. The Quality Index Method (QIM), which provides quantitative scores based on shrimp appearance, gill condition, meat texture, and odor, has become the mainstream sensory freshness evaluation method. Notably, previous studies have confirmed that ultra-low temperature storage effectively preserves sensory quality. Physical evaluation primarily relies on CIE color parameters, including lightness ($L^{*}$), redness/greenness ($a^{*}$), and yellowness/blueness ($b^{*}$), along with texture indicators to characterize the structural deterioration of shrimp meat [12,13,14]. In chemical evaluation, total volatile basic nitrogen (TVB-N), K-value and biogenic amines are core indicators for characterizing protein decomposition and spoilage, among which TVB-N is the most direct parameter to evaluate shrimp spoilage [15,16]. Microbiological detection complements the assessment system by monitoring the growth of dominant spoilage bacteria. Although the existing indicator and detection systems have been standardized, traditional off-line sampling and static detection methods fail to capture dynamic quality changes under cold chain temperature fluctuations, rendering them inadequate for real-time quality monitoring throughout the entire cold chain process.

With the rapid advancement of artificial intelligence, deep learning has emerged as a critical technical approach for aquatic product quality detection, time-series prediction, and intelligent grading, leveraging its superior capabilities in feature extraction and nonlinear fitting, thereby offering new solutions for intelligent cold chain quality management. Currently, deep learning algorithms such as CNN and the YOLO series have been widely applied in the intelligent evaluation of grains, fruits, vegetables, and aquatic products, with ongoing research focused on model lightweighting and accuracy optimization. Specifically, CNN-based detection systems with hardware acceleration enable high-precision and rapid identification of agricultural product quality, significantly enhancing detection automation [17,18]; an improved YOLOv8 model incorporating lightweight modules and attention mechanisms effectively addresses the issues of low detection accuracy and poor real-time performance in traditional fruit and vegetable disease detection, improving detection stability in complex scenarios [19,20]; a YOLOv5-based visual classification method automatically determines aquatic product freshness based on phenotypic features, overcoming the limitations of strong subjectivity and low efficiency associated with manual evaluation [21,22]; furthermore, existing reviews have systematically compared the application characteristics of CNN, YOLO, and R-CNN algorithms, clarifying current bottlenecks and future directions in intelligent grading technology, thereby providing theoretical support for intelligent evaluation research on agricultural products [23]. Overall, deep learning has matured in static image recognition and the quality classification of agricultural products, effectively compensating for the shortcomings of traditional detection methods. However, research on dynamic intelligent quality monitoring specifically targeting *L. vannamei* remains relatively underdeveloped. Current studies on shrimp quality control have largely focused on optimizing traditional preservation technologies, primarily through freezing process improvements [24,25], application of novel preservative packaging [26,27], and treatment with natural bio-preservative materials [28,29,30] to delay quality deterioration. These experiments have all been conducted under constant storage conditions with static physicochemical measurements, which only reflect terminal quality states under fixed conditions while overlooking the sustained impact of dynamic cold chain temperature fluctuations on the temporal evolution of shrimp quality. At present, research on deep learning-based multi-indicator time-series prediction, dynamic grading, and deterioration traceability for shrimp under fluctuating cold chain temperature conditions remains scarce, particularly regarding specialized time-series models tailored to the dynamic deterioration mechanisms in cold chains, which poses a challenge to meeting the requirements for precise and intelligent control throughout the entire aquatic cold chain process.

In summary, this study proposes a Tad-Transformer model for dynamic quality prediction and intelligent grading of *L. vannamei* under cold chain temperature fluctuation scenarios. By simulating realistic cold chain temperature fluctuation conditions and integrating multi-dimensional sensitivity analysis to screen high-sensitivity core quality indicators, a shrimp quality time-series dataset is constructed. Furthermore, an improved K-means++ clustering algorithm incorporating min-max constraints is employed to achieve adaptive quality grading. The proposed model enables simultaneous dynamic prediction of quality indicators and intelligent classification of deterioration levels, thereby providing reliable technical support for quality risk warning, deterioration traceability, and refined intelligent control in the aquatic cold chain.

## 2. Materials and Methods

### 2.1. Materials

Experimental samples were obtained from actual aquatic cold chain logistics scenarios. Live *L. vannamei* specimens with uniform body size, intact surfaces, good vitality, and no mechanical damage were selected for this study. Initial quality parameters of all samples were measured prior to storage treatment to establish experimental baselines. The quality indicators measured in the experiments were divided into chemical and physical categories. Chemical indicators included TVB-N and thiobarbituric acid reactive substances (TBARS), which were used to evaluate protein spoilage and lipid oxidation levels, respectively. Physical indicators included color parameters ($L^{*}$, $a^{*}$, $b^{*}$) and texture properties (hardness, springiness, resilience, chewiness), which were used to characterize the appearance, color, and structural changes of shrimp meat. Storage time and ambient temperature time-series data were simultaneously recorded during the experiments. After cleaning and draining, samples were sealed in sterile preservation boxes and pre-cooled at 4 °C for 24 h to eliminate the interference of initial temperature differences on the experimental results. For each temperature condition, three biological replicates, i.e., independent preservation boxes, were prepared. All quality indicators were measured in duplicate technical replicates, and the average value was used for subsequent analysis. At each sampling time point, three shrimps were randomly taken from each biological replicate to ensure representative sampling.

A completely randomized design was adopted for temperature treatments. The six temperature conditions, namely −40 °C, −18 ± 0.5 °C, −18 ± 2 °C, −18 ± 4 °C, −18 ± 6 °C, and −18 ± 8 °C, were randomly assigned to the preservation boxes. The measurement order for each indicator was also randomized to minimize systematic bias. The total storage period was 120 days, and sampling was conducted every 5 days, i.e., on days 0, 5, 10, 15, …, 120, yielding 25 sampling time points for each temperature group. Temperature fluctuations were continuously monitored throughout the entire storage period using temperature and humidity data loggers. These temperature gradients were selected to systematically cover the spectrum of thermal disturbances commonly encountered in commercial cold chains, ranging from stable storage to severe temperature abuse during transportation and transshipment. By analyzing the time-series patterns of various physicochemical and texture indicators of *L. vannamei* under different temperature conditions, a cold chain temperature–quality dynamic dataset was constructed.

TVB-N was determined using a UDK159 automatic Kjeldahl apparatus (VELP, Usmate, Italy) and expressed as mg/100 g. TBARS was measured using a Thermo Evolution 201 UV–Vis spectrophotometer (Thermo Scientific, Waltham, USA) and expressed as mg MDA/kg. Color parameters ($L^{*}$, $a^{*}$, $b^{*}$) were measured using an Ultrascan PRO benchtop spectrophotometer (Hunterlab, Reston, USA). Texture properties were analyzed using a TA.XT plus texture analyzer (Stable Micro System, Godalming, UK) with a P0.5 probe in texture profile analysis mode.

### 2.2. Multi-Dimensional Sensitivity Evaluation Method

Cold chain temperature fluctuation is the core factor inducing quality deterioration of *L. vannamei*. Different quality indicators exhibit significantly varying degrees of responsiveness to dynamic temperature changes. Screening temperature-sensitive core indicators is an important prerequisite for achieving dynamic quality evaluation and intelligent prediction of shrimp in cold chains. To accurately identify key quality parameters suitable for dynamic cold chain conditions, this study adopted a multi-dimensional sensitivity evaluation method that systematically quantified the sensitivity of various physicochemical and texture indicators with cold chain temperature fluctuations by combining time-series statistical characteristics, temperature correlation, and indicator variation amplitude.

The multi-dimensional sensitivity evaluation method used in this study mainly included linear regression analysis and temperature sensitivity analysis, which quantified the variation patterns of each quality indicator with storage time and cold chain temperature, thereby identifying key indicators that characterize shrimp quality deterioration and are sensitive to temperature fluctuations. First, preprocessing was performed on the complete time-series experimental dataset, including data cleaning and time-series normalization. Invalid interfering parameters were removed, and storage time, ambient temperature, and various quality indicators were retained as analysis variables. Second, univariate linear regression was used to fit the time-series deterioration rate of each indicator under different temperature fluctuation conditions. The regression slope was used to characterize the dynamic trend of the indicator with storage time, and variance testing was combined to ensure data validity. Finally, a multi-dimensional sensitivity scoring model was constructed by integrating the temperature dispersion characteristics, temperature correlation, and variation contrast of the indicators, as shown in the following formula:

(1) Score=0.4×std+0.4×ρ+0.2×R

where std represents the standard deviation of the indicator deterioration slopes under different temperature conditions, characterizing the discrete response of the indicator to temperature fluctuations; ρ represents the absolute value of the Pearson correlation coefficient between temperature and deterioration rate, quantifying the driving strength of temperature on quality changes; and R represents the slope contrast between different conditions, measuring the environmental discriminability of the indicator. Each weight was empirically assigned based on indicator contribution and normalized. The standard deviation and temperature correlation were assigned higher equal weights of 0.4 each, as they directly reflect the responsiveness of indicators to temperature variations, while the slope contrast was assigned a lower weight of 0.2 as a supplementary discrimination metric. A higher comprehensive score indicates that the indicator is more sensitive to cold chain temperature fluctuations and more effective in characterizing quality deterioration. This method effectively compensates for the limitations of single statistical analysis and accurately screens core quality indicators suitable for dynamic cold chain scenarios, providing core feature support for subsequent time-series dataset construction and intelligent model training.

### 2.3. Shrimp Quality Prediction Model Based on Tad-Transformer

#### 2.3.1. Overall Architecture and Principles of the Model

Based on the traditional Transformer network, this study integrated a dynamic time-varying coupling (TVC) graph and an advection-diffusion cooperative information propagation mechanism to construct the Tad-Transformer quality prediction model. This model no longer relies solely on self-attention for time-series modeling. Instead, it dynamically constructs a topological graph of time steps based on the time-series features of the samples, combines a graph convolutional network (GCN) to achieve local information diffusion, and simultaneously utilizes a global attention mechanism to accomplish information advection, ultimately achieving future quality prediction through a simplified decoder.

Compared with traditional mathematical fitting methods, Tad-Transformer can fully explore complex dependencies within long-term time-series data and capture correlation characteristics of various quality indicators via dynamic graph structures. Supported by the convection-diffusion collaboration mechanism, the model enables joint modeling of local and global temporal features synchronously, and supports dynamic rolling prediction. It can adapt to the dynamically changing environmental conditions during cold chain transportation, possessing superior generalization ability and engineering application value.

**Figure 1:**
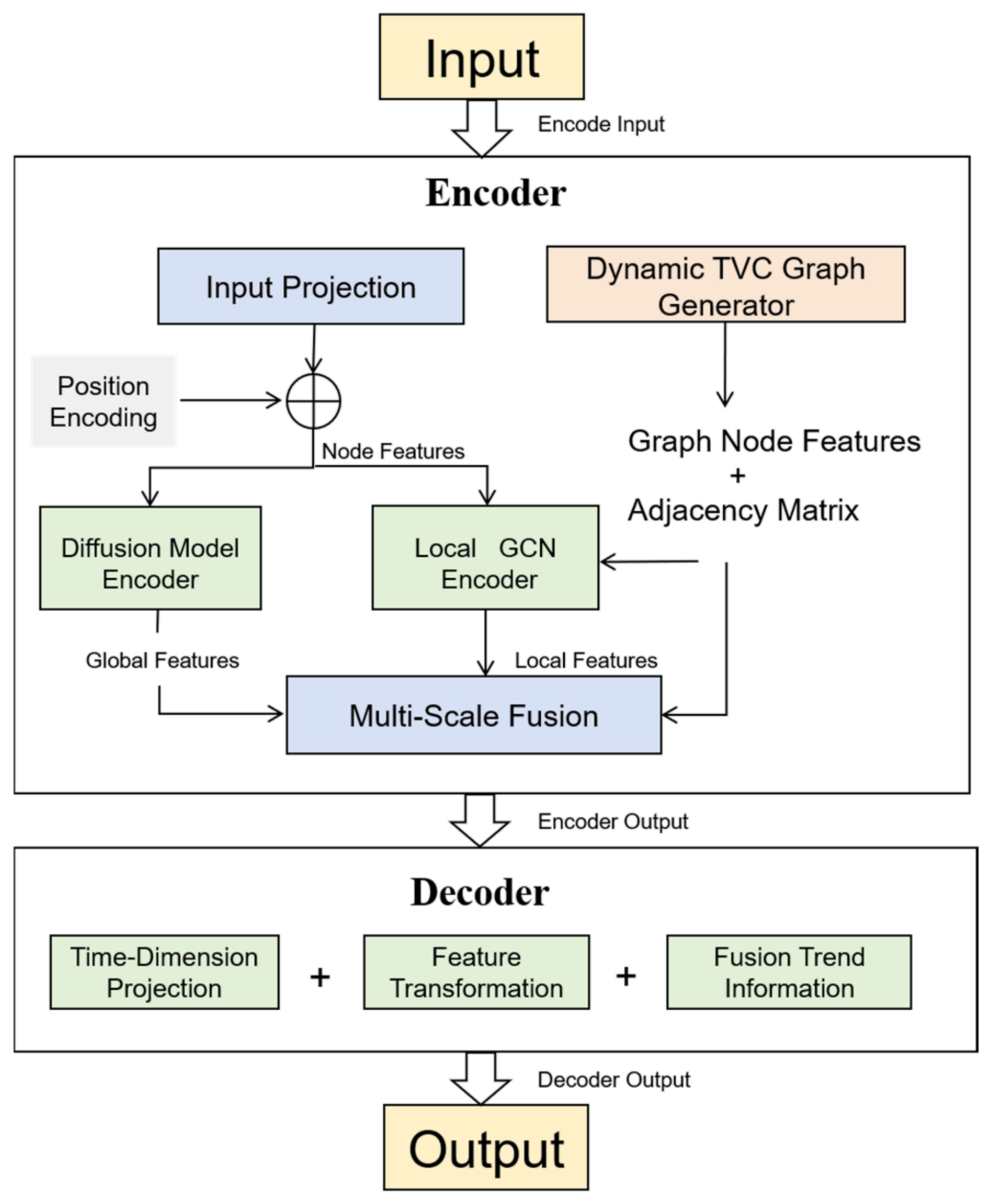
Tad-Transformer-based quality prediction model for *L. vannamei*.

The overall model consists of two parallel branches: node feature processing and dynamic TVC graph generation. It integrates local GCN, convection-diffusion Transformer and multi-scale feature fusion modules, and outputs prediction results combined with temporal trend terms. The overall workflow is illustrated in Figure 1. The input of the model is the time-series quality data of *L. vannamei* with the dimension format of [batch size, sequence length, feature dimension]. The mean trend term of the input sequence is calculated and initialized first, and then the sequence is fed into the encoder. The encoder outputs the dynamic TVC adjacency matrix and node feature matrix in parallel. Feature extraction is performed by the local GCN and convection-diffusion Transformer, respectively, followed by multi-scale fusion to obtain encoded features.

In the decoding stage, historical temporal dimensions are mapped to predicted temporal dimensions. Hidden layer features are acquired through feature transformation and trend fusion. Ultimately, predicted values are output via a fully connected projection layer, and the mean trend term of the original sequence is superimposed to obtain the shrimp quality prediction results for the next 7 days.

#### 2.3.2. Core Functional Modules

**Figure 2:**
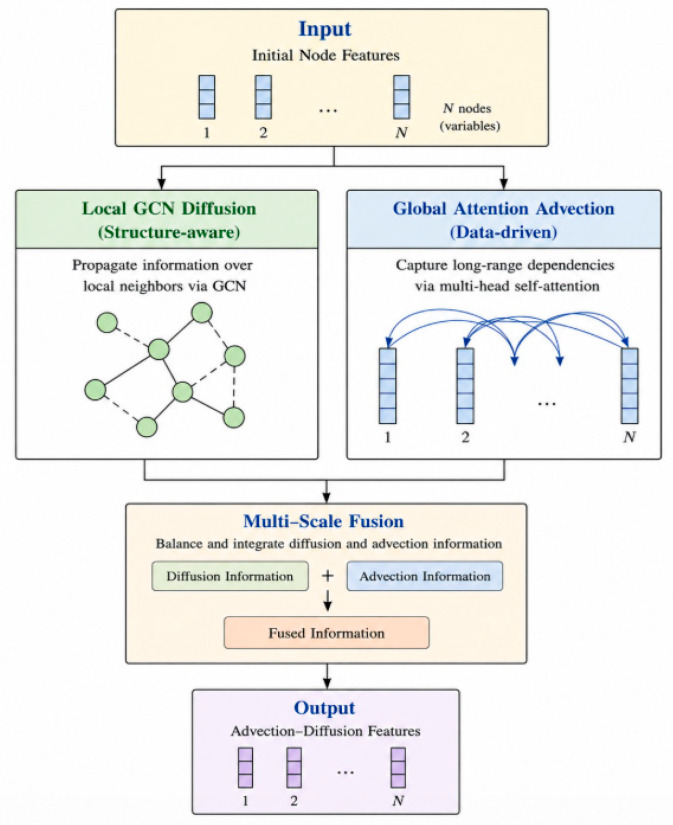
Convection-diffusion collaborative information propagation module.

The Tad-Transformer model consists of two core components: dynamic Time-Varying Coupled (TVC) graph generation and convection-diffusion collaborative information propagation. The two modules respectively implement local dynamic modeling and multi-scale global information interaction for cold chain time-series data, and work collaboratively to extract and model temporal quality features. The overall structure of these modules is presented in Figure 2.

First, the TVC graph generation module constructs an adaptive sparse adjacency matrix to describe the intrinsic correlations across different time steps and provide topological constraints for local information propagation. This procedure is implemented in three steps. Initially, the input time-series features are standardized, and the Pearson correlation coefficient matrix is calculated and adopted as the initial edge weights of the graph structure. Subsequently, diagonal elements of the matrix are removed. The Top-K strategy is utilized to select highly correlated time steps for constructing a sparse binary adjacency matrix, which is further supplemented with self-connections and symmetrized. Finally, a convolutional layer with symmetric kernels is applied to extract deep structural features of the graph. After dimensionality reduction and normalization, a dynamic adjacency matrix with the dimension of [B, 7, 7] is generated. Different from the fixed topology of conventional static graph models, the proposed structure updates adaptively along with variations in cold chain quality time series, and effectively characterizes the dynamic evolution of correlations among various indicators under temperature fluctuations.

Guided by the physical convection-diffusion theory, the collaborative information propagation mechanism integrates local diffusion via graph convolution and global convection via attention to achieve multi-scale interactive fusion of time-series information. The corresponding formula is defined as

(2) $$X_{k+1}=\beta \cdot GCN(X_{k})+(1-\beta )\cdot Attention(X_{k})$$

where β represents the diffusion coefficient, ranging from 0 to 1. In this study, β is set to 0.5 to balance the propagation weights of local and global information.

For the diffusion term, two cascaded GCN layers are built upon the TVC adjacency matrix to capture local temporal dependencies. The network adopts feature upsampling and downsampling structures, and residual connections are introduced to optimize model performance. The resulting local feature is $H_{local}\epsilon R^{81\times 32}$, and the calculation formula is given by

(3) $$H_{i}^{\left(l+1\right)}=\sigma \left(\sum_{j\epsilon N(i)}A_{ij}W^{\left(l\right)}H_{j}^{\left(l\right)}\right)$$

The convection term realizes global information interaction based on the multi-head attention mechanism. The features are first projected linearly and incorporated with positional encoding, and the correlation between different time steps is then calculated. The core formula is as follows:

(4) $$Attention(Q,K,V)=softmax(\frac{QK^{T}}{\sqrt{d_{k}}})V$$

where Q, K and V denote the query, key and value vectors, respectively, and $d_{k}$ is the attention dimension, set to 4 in this work. Residual connections are also employed in this branch, and the obtained global feature is $H_{global}\epsilon R^{N_{v}\times 6\times 32}$. To fully exploit multi-scale temporal features, the information propagation process is expanded into four orders. Features derived from each order are concatenated and fused, which enhances the model’s capability to fit complex temporal variations in cold chain data.

After acquiring three types of temporal representations including graph node features, local diffusion features and global convection features, the model proceeds with processing via the multi-scale fusion, decoding and prediction module. Adaptive learnable weights are assigned to different features, and multi-scale feature fusion is realized through weighted summation, linear transformation and layer normalization to produce unified deep encoded features. When the fused features are fed into the decoder, temporal dimension conversion is conducted first, and hidden prediction features are obtained via a two-layer feedforward network and normalization. Temporal trend features are mapped to the corresponding dimensions for further fusion, and the mean trend term of the input sequence is superimposed to correct prediction errors. Ultimately, a fully connected layer performs feature projection and outputs the predicted shrimp quality values for future periods.

#### 2.3.3. Data Partition and Experimental Setup

We adopted a chronological split for the dataset. For each temperature condition, the first 17 time points covering days 0 to 80 were used as the training set, the next 4 time points covering days 85 to 100 as the validation set, and the last 4 time points covering days 105 to 120 as the test set, ensuring that all training samples preceded the validation and test samples in time. The training set was used for model optimization, the validation set for hyperparameter tuning and early stopping to prevent overfitting, and the test set for final performance evaluation. Standard K-fold cross-validation with random shuffling was not employed, as it would break the temporal dependencies inherent in time-series data and lead to data leakage. In addition, the model adopts a rolling prediction strategy, wherein the most recently observed real data are incorporated into the input sequence at each prediction step to enable dynamic updates of the model state. All compared models, including Transformer, Informer, and FEDformer, were trained and evaluated under the identical data partition to ensure fairness. To avoid data leakage across temperature conditions, all samples from each temperature group were assigned exclusively to the training, validation, or test sets according to the same chronological rule, without mixing samples from different temperature conditions within the same set.

### 2.4. Quality Grading and Evaluation of L. vannamei

To realize automatic quality grading of *L. vannamei* during cold chain circulation, this study proposes an improved K-means++ clustering algorithm. By introducing the max-min sample constraint rule and a probability-weighted initial cluster center selection strategy, an intelligent clustering model suitable for multi-dimensional quality indicators is established. Adaptive quality grading of shrimp is implemented based on the screened core quality indicators.

#### 2.4.1. Data Preprocessing

Raw quality data suffer from outliers, inconsistent dimensions and divergent variation trends of indicators. Therefore, standard preprocessing is required prior to clustering to guarantee reliable model inputs.

In this study, the box plot method was adopted to detect outliers. The lower quartile (Q1) and upper quartile (Q3) of each indicator were calculated, and the interquartile range (IQR) was defined as IQR = Q3 − Q1. Samples outside the interval [Q1 − 1.5 IQR, Q3 + 1.5 IQR] were identified as outliers and replaced with the median of the corresponding indicator to reduce the interference of abnormal samples on the iteration of cluster centers.

Min-max normalization was applied to eliminate dimensional differences and map all data to the range [0, 1]. The formula is expressed as

(5) $$x_{i,j}^{\prime }=\frac{x_{i,j}-x_{min,j}}{x_{max,j}-x_{min,j}}$$

where $x_{i,j}$ denotes the original value of the j-th indicator for the i-th sample, $x_{min,j}$ and $x_{max,j}$ are the minimum and maximum values of the j-th indicator, respectively, and $x_{i,j}^{\prime }$ represents the normalized value. After normalization, all indicators are assigned equal weights in the clustering process.

Shrimp flesh hardness is positively correlated with product quality, while TVB-N, TBARS and $b^{*}$ value rise gradually as quality deteriorates, leading to inconsistent variation trends among indicators. To unify the changing trends of all indicators, a reverse transformation is performed on the normalized hardness values:

(6) $${Hardness}_{adj}=1-{Hardness}_{std}$$

where ${Hardness}_{std}$ is the normalized hardness value, and ${Hardness}_{adj}$ is the adjusted hardness feature.

#### 2.4.2. Max-Min Rule

This algorithm integrates the probability-weighted initial center selection strategy with the max-min rule. On one hand, it optimizes the distribution of initial cluster centers and reduces the risk of the algorithm falling into local optima. On the other hand, it imposes constraints on sample assignment to improve the discrimination of different quality grades and the stability of clustering results.

In this study, Euclidean distance is used to measure the similarity between samples and cluster centers. Let the sample set be $X=\left\{x_{1},x_{2},\ldots ,x_{n}\right\}$ and the cluster center set be $C=\left\{C_{1},C_{2},\ldots ,C_{k}\right\}$. The Euclidean distance from sample $x_{i}$ to the k-th cluster center $C_{k}$ is defined as

(7) $$d(x_{i},C_{k})=|{|x}_{i}-C_{k}||$$

The quantity constraint for samples in a single cluster is formulated as follows:

(8) $$L\leq |C_{k}|\leq U$$

where L and U denote the minimum and maximum allowable number of samples within a single cluster, respectively, and |$C_{k}$| represents the total number of samples in the k-th cluster.

Sample assignment follows the distance priority principle strictly. Each sample is first allocated to the nearest cluster center to ensure compact feature distribution within clusters. If the target cluster reaches its upper limit of sample capacity, the sample will be assigned to the second nearest cluster that is not fully occupied. When the number of samples in a cluster is lower than the preset lower bound, samples with the minimum migration cost are selected from clusters with sufficient samples for supplementation. This constraint mechanism prevents the formation of extreme clusters and yields well-defined and evenly distributed clusters corresponding to high-, medium- and low-quality grades.

#### 2.4.3. Algorithm Implementation Procedures

**Figure 3:**
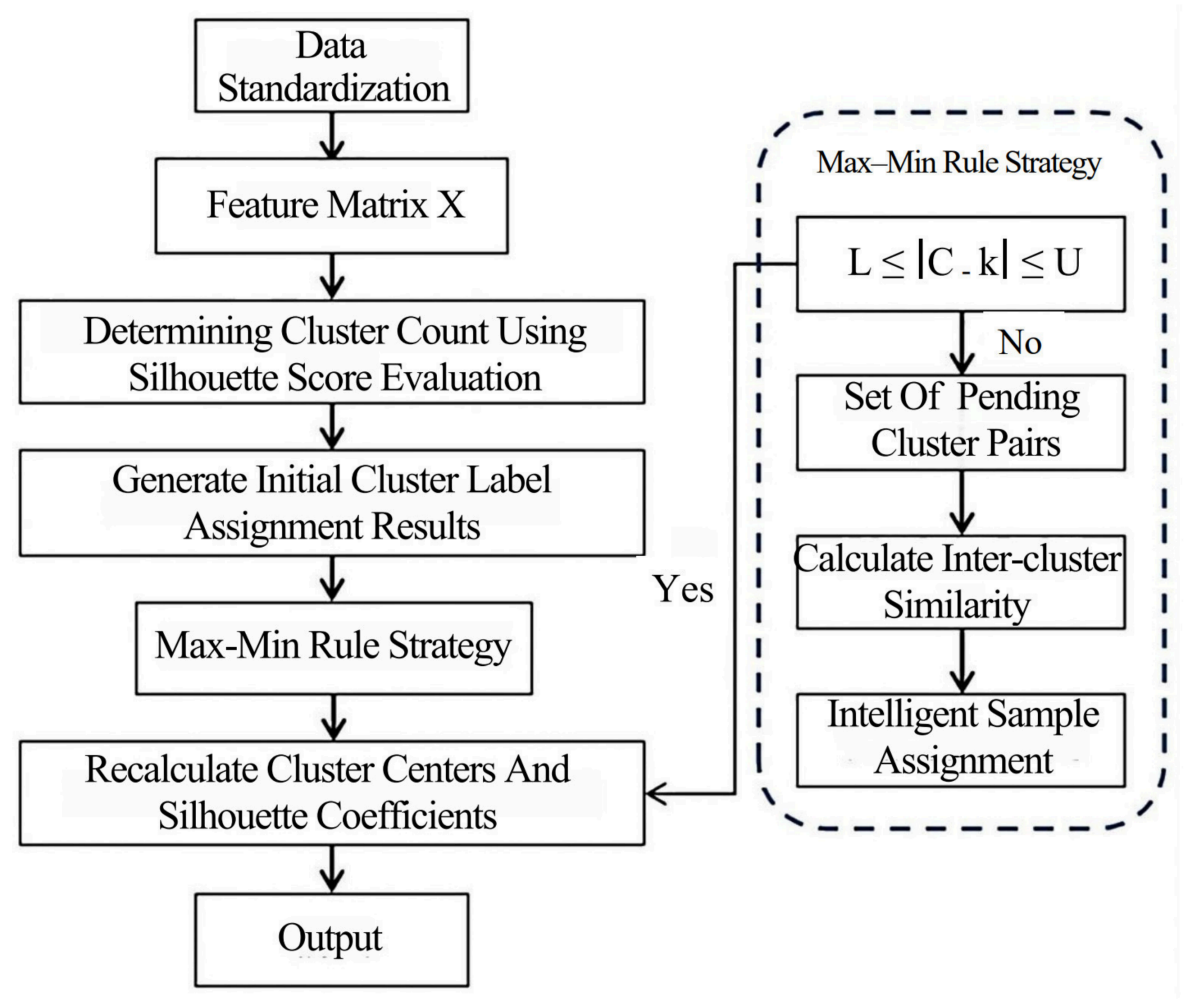
Flowchart of the K-means++ algorithm based on the max-min rule.

The overall workflow of the proposed clustering model is illustrated in Figure 3, and the detailed implementation steps are described as follows.

First, four indicators including TVB-N, TBARS, $b^{*}$ and flesh hardness are imported. Outliers are eliminated using the interquartile range method, and all data are then scaled uniformly to the range of [0.1, 1.0] via the Min-Max normalization.

Subsequently, the hardness indicator is reversely adjusted according to Equation (6) to unify the variation trends of all four indicators.

The number of clusters k is set sequentially from 2 to 10. Clustering is performed by combining the K-means++ algorithm and the max-min rule. The silhouette coefficient is adopted to comprehensively evaluate intra-cluster compactness and inter-cluster separation, so as to determine the optimal number of clusters.

Sample assignment is conducted based on Euclidean distance and sample quantity constraints. The cluster centers are updated iteratively until the clustering results converge.

Finally, the optimal cluster number is determined as k = 3. Quality grades are divided according to the mean values of cluster centers in the normalized space. The cluster with the minimum mean value corresponds to high quality, the one with the intermediate mean value represents medium quality, and the cluster with the maximum mean value corresponds to low quality.

## 3. Results

### 3.1. Dataset Construction and Core Quality Indicator Selection

#### 3.1.1. Experimental Design and Data Collection

According to the actual storage and transportation conditions for aquatic product cold chains, six groups of gradient temperature conditions were set to simulate frozen storage and transportation processes, namely −40 °C, −18 ± 0.5 °C, −18 ± 2 °C, −18 ± 4 °C, −18 ± 6 °C and −18 ± 8 °C. The total storage period lasted for 120 days.

During the whole experiment, ambient temperature and storage time were continuously monitored. The detected indicators included physicochemical and texture parameters, such as TVB-N, TBARS, color parameters ($L^{*}$, $a^{*}$, $b^{*}$), hardness, springiness, chewiness and resilience. A temperature and humidity logger was used to collect environmental parameters in real time. Sampling and indicator measurement were performed at regular time intervals. Finally, a time-series quality dataset of *L. vannamei* tailored for cold chain scenarios was established.

#### 3.1.2. Evaluation of Temperature Sensitivity of Quality Indicators

**Figure 4:**
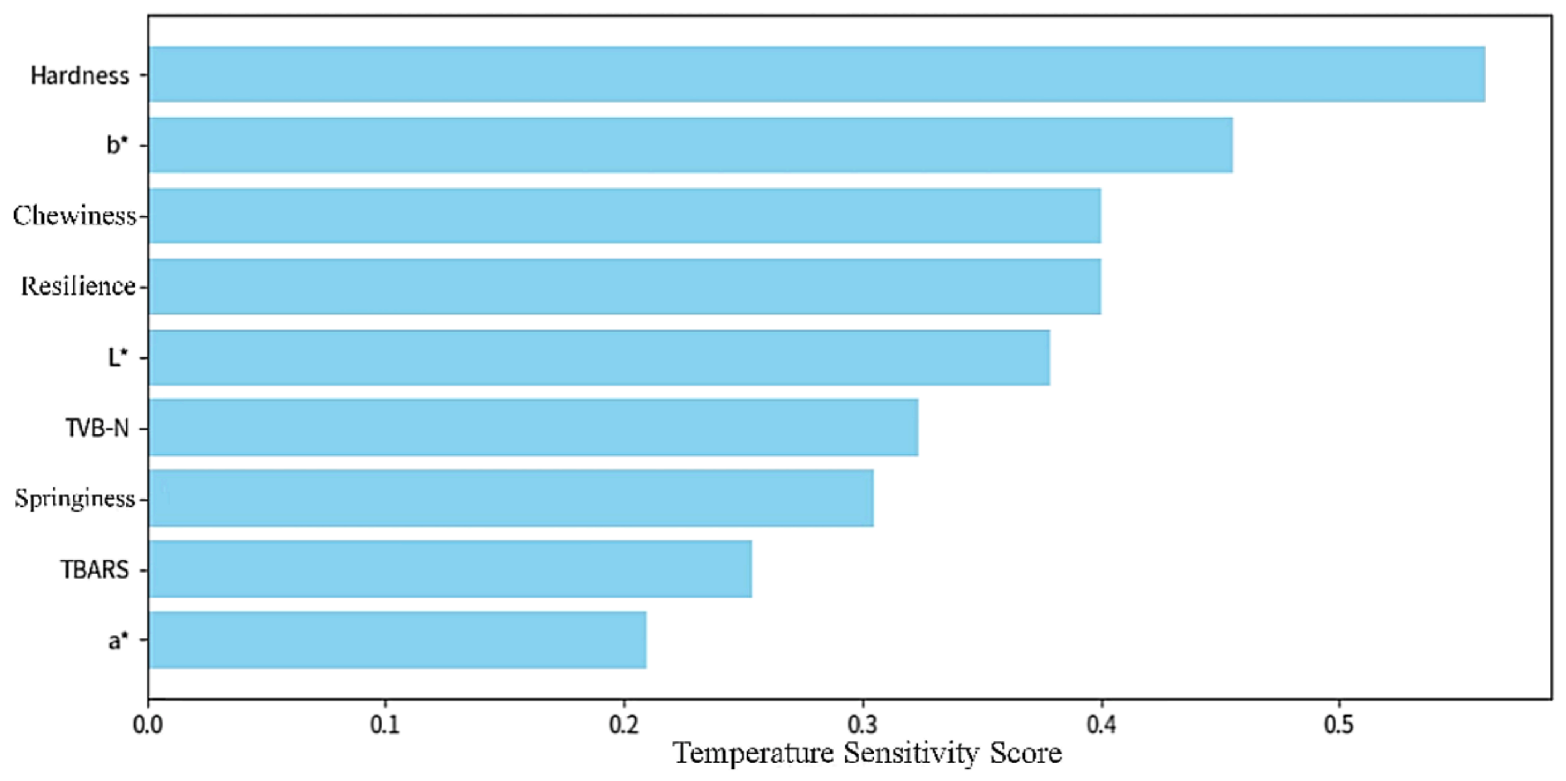
Sensitivity scores of quality indicators for *L. vannamei*.

The temperature sensitivity scores of each quality indicator are presented in Figure 4. It can be observed that hardness and yellowness $b^{*}$ achieve higher scores, indicating the strongest sensitivity to temperature fluctuations during cold chain logistics.

Combined with the visualization results, a refined quantitative ranking was conducted. The comprehensive ranking of temperature sensitivity for all indicators is listed in Table 1. Comparative analysis reveals that hardness and yellowness $b^{*}$ show the most remarkable responses to temperature fluctuations.

Considering the physicochemical implications, characterization dimensions and practical cold chain application requirements, four indicators including hardness, $b^{*}$, TVB-N and TBARS were finally selected as the core parameters for subsequent modeling and analysis. Specifically, hardness and $b^{*}$ characterize the texture and surface color of shrimp, while TVB-N and TBARS reflect the degrees of protein degradation and lipid oxidation, respectively. These four indicators can comprehensively describe the quality deterioration of *L. vannamei* from physical, chemical and visual perspectives. The four selected indicators are closely linked to the physiological processes underlying shrimp spoilage. Hardness reflects the degradation of myofibrillar proteins and breakdown of muscle cell integrity, driven mainly by endogenous proteases and collagenases. TVB-N is a classical marker of protein decomposition, produced through microbial deamination of amino acids. TBARS measures secondary lipid oxidation products, which compromise membrane integrity and contribute to off-flavor development. The increase in $b^{*}$ is associated with enzymatic browning and pigment changes, partly mediated by polyphenol oxidase activity and interactions between lipid oxidation products and amino groups. Regarding the excluded texture parameters, springiness, resilience, and chewiness were omitted despite their relatively high sensitivity scores because they are more sensitive to sample heterogeneity—particularly muscle fiber orientation and preparation procedures—and show greater measurement variability than hardness. For practical cold chain monitoring, we prioritized indicators that combine sensitivity with robustness and ease of routine measurement.

#### 3.1.3. Temporal Variation Characteristics of Core Indicators

**Figure 5:**
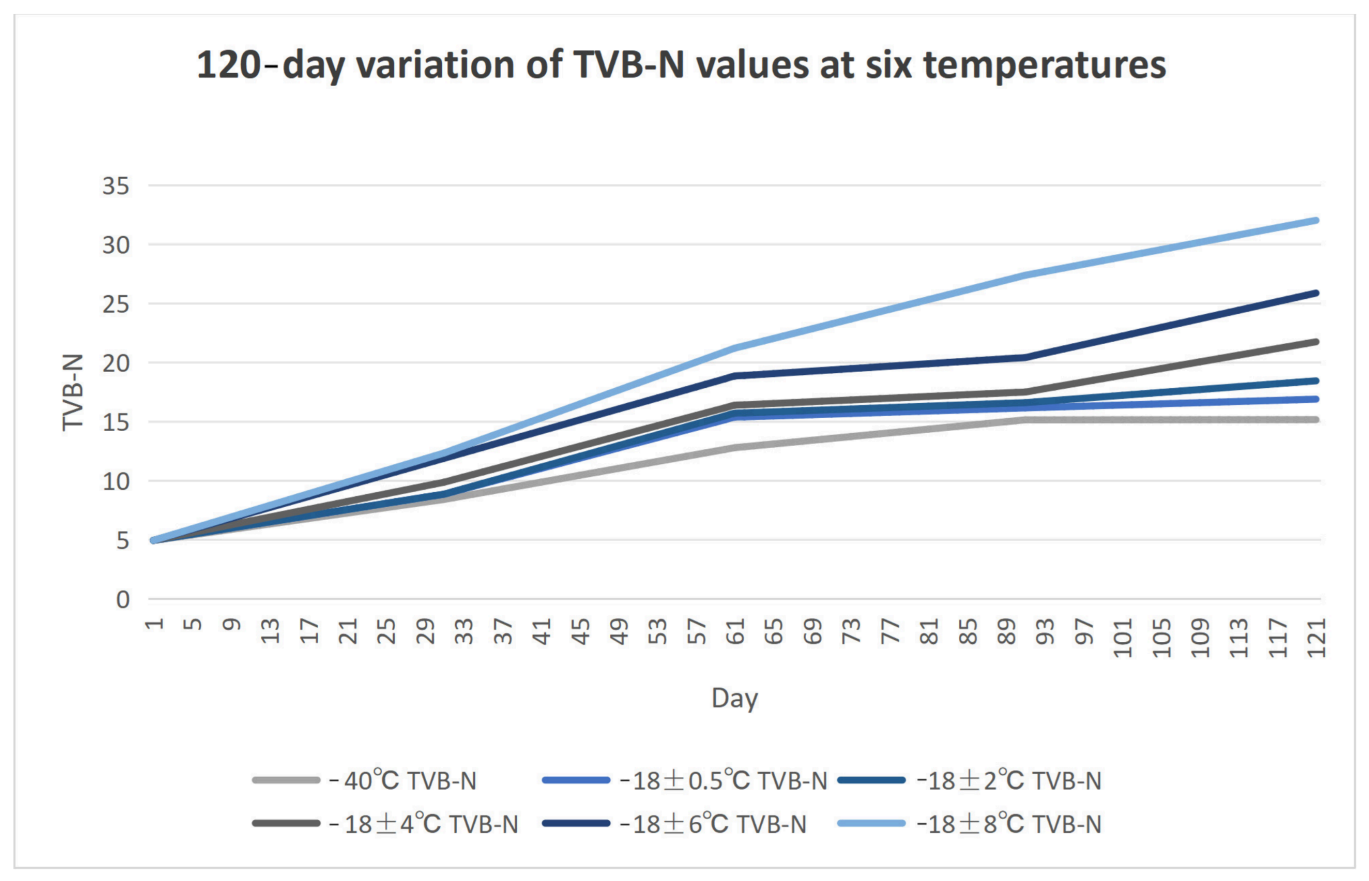
Changes in TVB-N values of *L. vannamei* under different temperatures.

**Figure 6:**
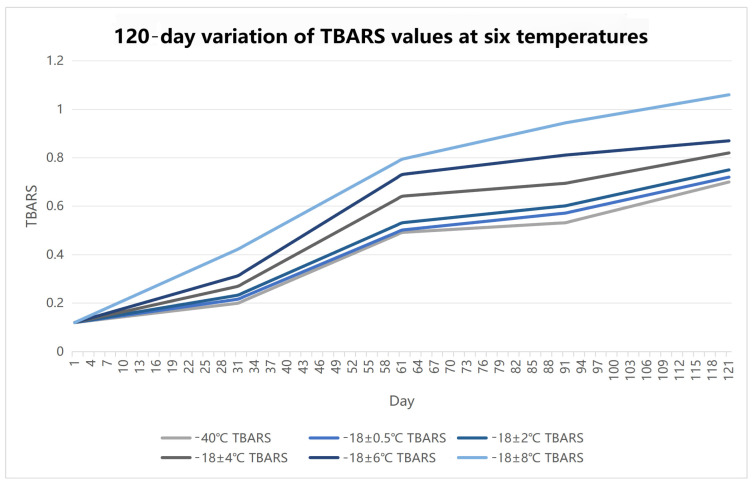
Changes in TBARS values of *L. vannamei* under different temperatures.

**Figure 7:**
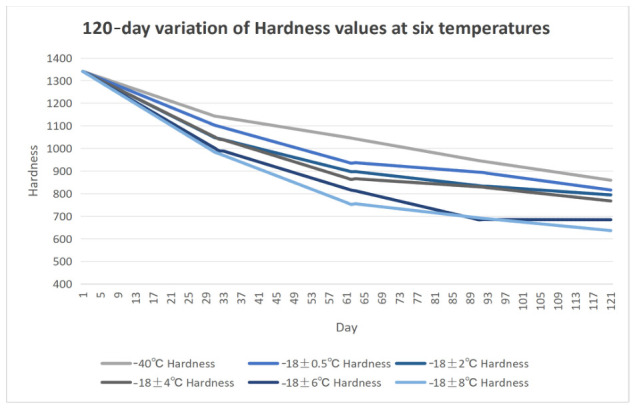
Changes in hardness values of *L. vannamei* under different temperatures.

**Figure 8:**
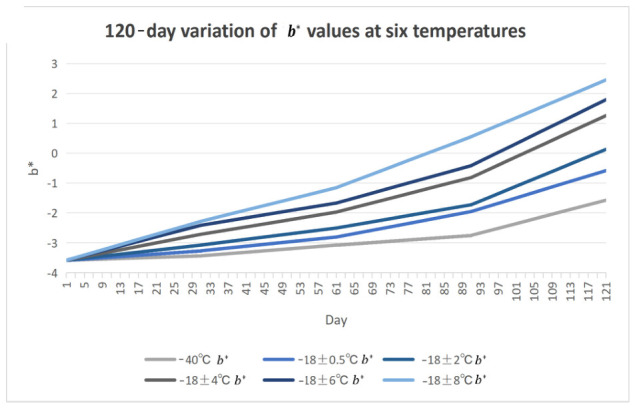
Changes in $b^{*}$ values of *L. vannamei* under different temperatures.

Using the selected evaluation indicator system, time-series datasets under six temperature conditions were established by simulating typical temperature fluctuations and ultra-low temperature storage environments in cold chains. The experimental temperatures were set as −18 ± 0.5 °C, −18 ± 2 °C, −18 ± 4 °C, −18 ± 6 °C, −18 ± 8 °C and −40 °C. The datasets record the dynamic variations in the four core indicators throughout the 0–120 days of storage. The variation trends of each indicator are presented in Figure 5, Figure 6, Figure 7 and Figure 8. In these figures, the horizontal axis represents storage days, and the vertical axis denotes the corresponding indicator values. Curves in different colors correspond to different temperature groups.

It is observed that microbial reproduction, endogenous enzyme activity, protein degradation and lipid oxidation are strongly suppressed at −40 °C ultra-low temperature. All four core indicators change slightly, demonstrating an optimal preservation effect.

Mechanistically, rising TVB-N values correspond to protein spoilage in shrimp muscle; increased TBARS levels indicate lipid oxidation and off-flavor formation. A higher $b^{*}$ value is associated with shrimp yellowing induced by enzymatic reactions and microbial activities, while decreased hardness means muscle tissue damage and flesh softening caused by freezing and temperature fluctuations.

The four indicators can sensitively respond to cold chain temperature disturbances and fully characterize the quality deterioration of *L. vannamei*. The established time-series dataset with multiple temperature gradients provides reliable data support for the training, testing and validation of subsequent deep learning models.

### 3.2. Experimental Results and Analysis of the Tad-Transformer Quality Prediction Model

#### 3.2.1. Experimental Environment and Model Parameter Settings

Model construction, training and testing were implemented based on the PyTorch deep learning framework. The hardware and software configurations for the experiments are listed in Table 2. Key hyperparameters of the model were set uniformly, and the detailed values are presented in Table 3. To objectively evaluate model performance, conventional Transformer, Informer and FEDformer were selected for comparative analysis.

#### 3.2.2. Model Evaluation Metrics

Common metrics for time-series forecasting were adopted to quantify model accuracy, including Mean Absolute Error (MAE), Mean Squared Error (MSE), Root Mean Squared Error (RMSE), Mean Absolute Percentage Error (MAPE) and Mean Squared Percentage Error (MSPE). MAE, MSE and RMSE measure the absolute deviation between predicted and actual values. MAPE and MSPE eliminate the influence of dimensional differences and characterize relative prediction errors. Smaller values of all error metrics indicate higher prediction accuracy.

These five metrics collectively provide a comprehensive evaluation of model performance from complementary perspectives. MAE reflects the average absolute prediction error and is insensitive to outliers, making it a straightforward indicator of overall prediction accuracy. MSE and RMSE amplify larger deviations by squaring the errors and are therefore more sensitive to extreme prediction errors, serving as indicators of worst-case prediction risk; RMSE is particularly interpretable, as it shares the same scale as the original data. MAPE expresses the error as a relative percentage, eliminating scale differences across indicators with different units and enabling consistent comparisons across temperature conditions or quality metrics. MSPE emphasizes the relative proportion of large errors and is sensitive to anomalous predictions, effectively reflecting model performance under extreme error scenarios. The combined use of these metrics allows for a comprehensive assessment of prediction accuracy, robustness against outliers, and the risk of extreme deviations. When the proposed model consistently outperforms baseline models across most of these metrics under all temperature conditions, this indicates that it achieves superior overall accuracy, stability, and generalization capability in dynamic cold chain scenarios.

#### 3.2.3. Comparative Analysis of Prediction Results Among Different Models

Taking the core indicator $b^{*}$ as an example, the prediction errors of all models under six cold chain temperature conditions are presented in Table 4. The results show that the proposed Tad-Transformer achieves distinctly lower MAE, MSE, RMSE, MAPE and MSPE values than all comparative models across all temperature scenarios, demonstrating superior prediction performance. $b^{*}$ was selected as the representative example because it exhibits the largest variation among the quality indicators during frozen storage and is most critical to color evaluation. The complete prediction errors for the other quality indicators, including TVB-N, TBARS, and Hardness, are provided in Appendix A, Table A1, Table A2 and Table A3.

Among them, MAE reflects the average absolute deviation between predicted and actual values, intuitively representing the magnitude of prediction error—the smaller the MAE, the better the model’s performance. MSE is a commonly used metric in statistical analysis to measure the error between original data and predictions; it is a simple yet effective tool that clearly reflects prediction accuracy. RMSE measures the average level of deviation between predicted and actual values, also reflecting overall prediction accuracy. MAPE has a clear criterion: a value of 0% indicates a perfect model, while a MAPE exceeding 100% indicates a poor-quality model. MSPE primarily measures the squared proportional error of predictions relative to actual values, emphasizing the relative proportion of the error.

The results are shown in Table 4. Taking indicator $b^{*}$ as an example, the maximum values of MAE, MSE, RMSE, MAPE, and MSPE for the Tad-Transformer model under different temperature conditions are 0.045506, 0.003814, 0.061759, 0.061062, and 0.008537, respectively. All of these maximum values are lower than the minimum values of the corresponding metrics for other comparison models under the same conditions, demonstrating that the Tad-Transformer model achieves the highest prediction accuracy and significantly outperforms the other models.

To assess the statistical robustness of the reported improvements, all models were trained and evaluated using five different random seeds, namely 42, 1234, 2024, 3407, and 9999. For each seed, the data split was fixed to ensure comparability across runs. The mean and standard deviation of each error metric were calculated across the five runs and are summarized in Table 5. The standard deviations of the proposed Tad-Transformer are consistently smaller than those of the comparison models across all metrics, confirming that the observed performance improvements are statistically robust and not attributable to random initialization.

These results confirm that the proposed Tad-Transformer not only achieves superior prediction accuracy but also exhibits consistently higher stability across different random initializations compared with the baseline models.

### 3.3. Results and Analysis of Quality Grading Based on Improved K-Means++ Algorithm

#### 3.3.1. Experimental Results for Selection of Optimal Cluster Number

The silhouette coefficient is adopted in this experiment to evaluate the clustering performance, which ranges from −1 to 1. A value closer to 1 indicates higher intra-cluster sample compactness and better inter-cluster discrimination. The number of clusters k is set from 2 to 10. Comparative tests are conducted on the traditional K-means algorithm, the original K-means++ algorithm, and the improved K-means++ algorithm integrated with the max-min rule.

**Figure 9:**
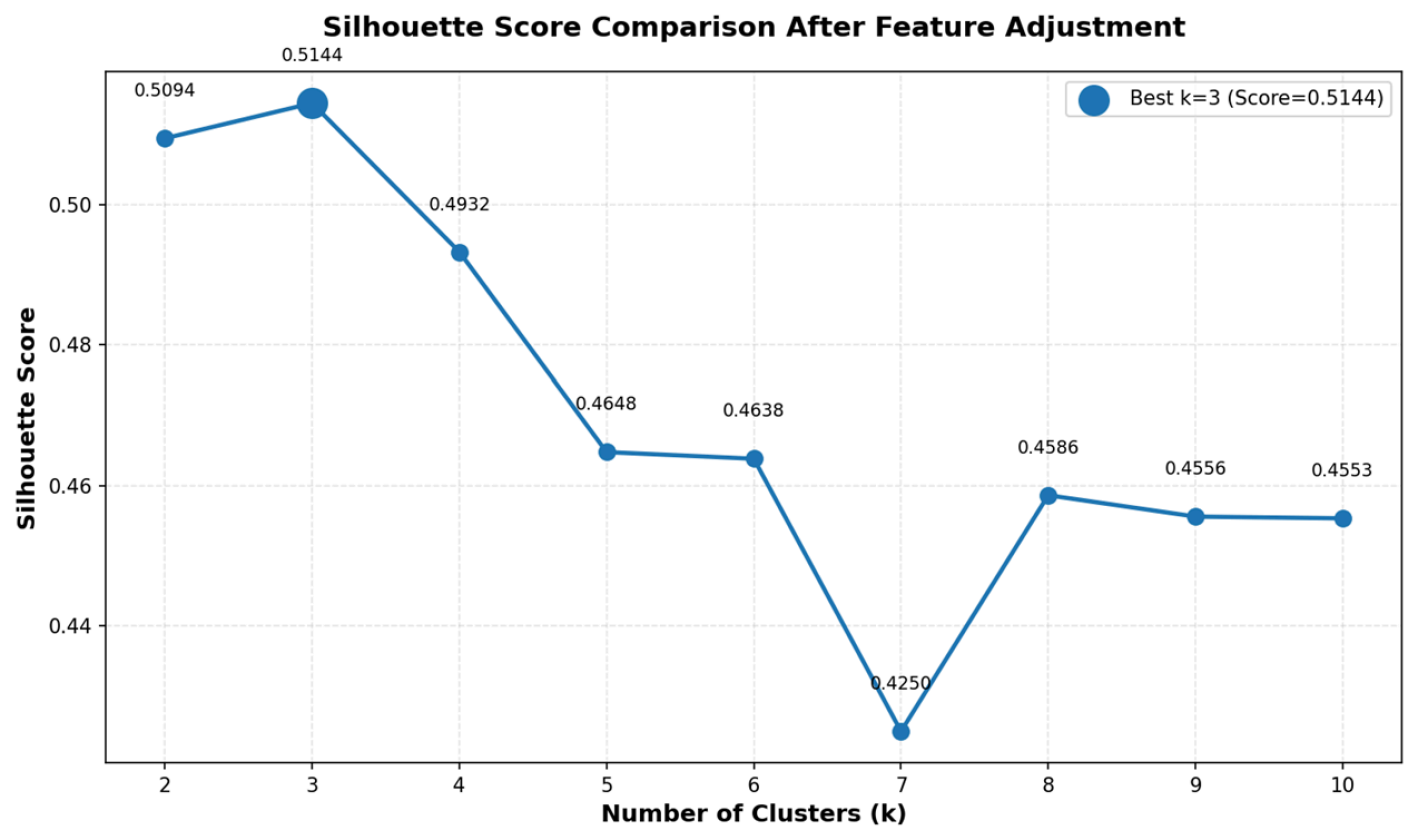
Comparison of silhouette coefficients under different cluster numbers.

The variation in silhouette coefficients of each algorithm under different cluster numbers is presented in Figure 9. The experimental results show that the improved K-means++ algorithm achieves the maximum silhouette coefficient of 0.5144 when k = 3, corresponding to the optimal clustering performance. Accordingly, the quality of *L. vannamei* is classified into three grades: high quality, medium quality and low quality.

Table 6 presents the statistical silhouette coefficients of different algorithms under typical cluster numbers. The comparison reveals that the improved algorithm proposed in this paper yields higher silhouette coefficients across all cluster numbers than the traditional K-means and original K-means++, with distinctly enhanced clustering stability and discrimination capability.

**Figure 10:**
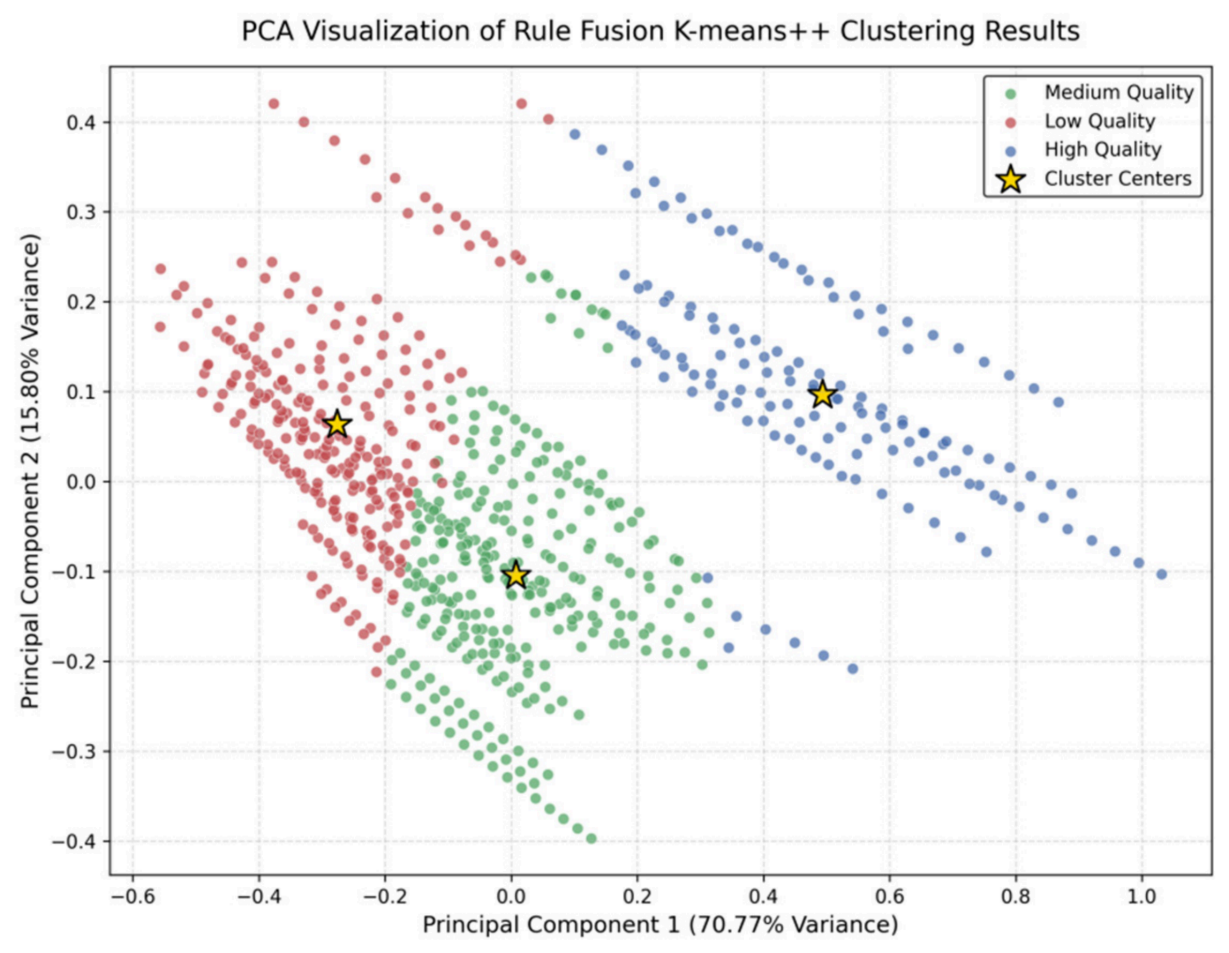
PCA scatter plot of clustering results by rule-fused K-means++.

To intuitively demonstrate the clustering distribution of samples, principal component analysis (PCA) is used to reduce the four-dimensional quality features to a two-dimensional space for visualization. The results are shown in Figure 10. It can be observed that samples of the three quality grades have clear boundaries and regular distribution in the feature space, which further verifies the rationality of k = 3 and the effectiveness of the improved clustering algorithm.

#### 3.3.2. Classification Results and Characteristic Analysis of Quality Grades

Clustering was performed on the sample data established in this experiment to obtain the cluster centers, sample quantities and proportions of each quality grade. The statistical results are presented in Table 7. Combined with the physicochemical implications of each indicator, the characteristics of the three quality grades are analyzed and discussed.

The experimental results indicate that high-quality samples have the lowest values of TVB-N, TBARS and $b^{*}$ among the three groups, which means the shrimp exhibits the mildest protein degradation, lowest lipid oxidation and least yellowing. Since the clustering analysis adopted the inversely transformed hardness values, this group has a hardness value of 0.4004, the minimum of all groups. It corresponds to the highest original flesh hardness, presenting firm texture and optimal structural properties with the overall best quality.

For medium-quality samples, all four indicators stay at moderate levels. No obvious deterioration occurs in protein degradation, lipid oxidation, surface color and flesh texture, making shrimp of this grade the mainstream commodity in the market.

Low-quality samples show remarkably increased TVB-N and TBARS values, indicating severe spoilage and lipid oxidation. The high $b^{*}$ value reflects serious yellowing of shrimp bodies. Its transformed hardness reaches the maximum value of 0.5218, which corresponds to a sharp decline in original flesh hardness and obvious softening, resulting in nearly lost commercial and edible value.

In terms of sample distribution, low-quality samples account for the largest proportion at 44.7%, while high-quality samples represent the smallest share at 16.7%. This distribution conforms to the natural quality deterioration rule of *L. vannamei* during long-term cold chain storage.

To better understand the practical meaning of the three clusters, we compared them with established quality benchmarks. The TVB-N threshold of 30 mg/100 g was used as the reference, as this value is widely recognized for fresh marine shrimp. In our data, the high-quality cluster stayed well below this limit during the first 60 days of storage, while the low-quality cluster exceeded it after day 80. For hardness, $b^{*}$, and TBARS, no unified regulatory limits exist, but their trends closely tracked the TVB-N changes, reinforcing the clustering validity. The three grades can be translated into clear supply-chain decisions: high-grade shrimp can be directed to premium markets, medium-grade products should be prioritized for fast distribution given their limited remaining shelf life, and low-grade items should be removed from the retail chain and redirected to processing. This framework allows logistics managers to take timely actions, such as adjusting temperature settings or re-routing shipments based on the predicted grade.

#### 3.3.3. Grade Matching and Accuracy Verification of Prediction Results

Taking the three cluster centers obtained from clustering of actual samples as the unified evaluation criterion, Euclidean distance was used to match the quality grades predicted by different models. Precision (P), Recall (R) and F1-score were selected as evaluation metrics to quantify the grading and prediction performance of each model. The test results are shown in Table 8.

As shown in the experimental results, the F1-scores of Tad-Transformer for low-, medium- and high-quality grades are 90.85%, 94.23% and 91.48%, respectively. It achieves the highest overall prediction accuracy with minor metric fluctuations across different grades, and its stability is remarkably superior to that of other comparative models. Transformer ranks second, while FEDformer and Informer deliver relatively lower overall grading accuracy and lack sufficient capability to identify and distinguish borderline-quality samples. The comprehensive results verify that Tad-Transformer can simultaneously realize numerical prediction of quality indicators and automatic grade classification for *L. vannamei*, which meets the practical requirements of online monitoring, quality grading and risk early warning in aquatic product cold chains.

## 4. Discussion

Cold chain temperature fluctuation is the key factor leading to quality degradation of *L. vannamei*, and dynamic quality prediction and intelligent classification play an important role in advancing the aquatic industry. Four core indicators with high temperature sensitivity were selected through sensitivity analysis. The constructed indicator system comprehensively reflects the texture, color and chemical deterioration of shrimp during storage, and its time-varying trends conform to classic theories of aquatic preservation.

Aiming at the nonlinear and volatile characteristics of cold chain time-series data, the Tad-Transformer model combining graph convolution and advection-diffusion mechanisms was proposed. It outperforms traditional Transformer and other prevalent time-series models in prediction accuracy and robustness across different temperature scenarios. The improved K-means++ algorithm overcomes the limitations of traditional clustering approaches. The three quality grades are well distinguished, and the model performs well in data prediction and grade matching for practical monitoring.

This study has several limitations. The experiments were conducted under controlled laboratory conditions, which cannot fully replicate the complexity of real cold chain operations. Mechanical vibration, compression stacking, and intermittent temperature spikes during loading and unloading, for instance, were not explicitly considered. Also, our model was trained exclusively on *L. vannamei* from a single source, and whether it generalizes to other shrimp species or production batches is unknown. Packaging systems also matter: vacuum packaging or modified atmosphere packaging, which are common in commercial practice, can substantially alter bacterial growth and chemical reaction kinetics, potentially affecting the model’s performance. Similarly, while a 120-day storage period covers typical long-term frozen logistics, shorter or extended durations may yield different quality evolution patterns.

Regarding the model’s applicability to real cold chain scenarios, we acknowledge that field conditions often involve more irregular temperature fluctuations and less standardized measurements than controlled laboratory experiments. Nonetheless, the proposed Tad-Transformer is expected to exhibit reasonable robustness in such environments. First, the temperature conditions in our experiments already cover a wide range from −40 °C to −18 ± 8 °C, which spans most thermal disturbances encountered in commercial cold chains. Second, the self-attention mechanism inherently captures long-range dependencies, making the model less sensitive to irregular input sequences. Third, the pointwise prediction strategy does not rely on strict temporal regularity, which reduces the impact of non-uniform measurement intervals. However, we fully agree that further validation under real field conditions is necessary.

Future work will address these gaps by validating the model across multiple batches, packaging formats, and real cold chain environments using IoT-based monitoring data.

## Figures and Tables

**Table 1:** Ranking of comprehensive temperature sensitivity index.

Rank	Indicator	Sensitivity Score	Average Slope	Temperature Correlation
1	Hardness	0.561	4.5406	0.2196
2	$b^{*}$	0.4557	0.0322	0.3952
3	Chewiness	0.4	0.0014	0.4104
4	Resilience	0.3999	0.0014	0.4102
5	$L^{*}$	0.3787	0.0537	0.4165
6	TVB-N	0.3239	0.1422	0.1367
7	Springiness	0.3046	0.0019	0.1366
8	TBARS	0.2539	0.0062	0.1559
9	$a^{*}$	0.2098	0.0182	0.0213

**Table 2:** Experimental environment configuration.

Environment Type	Item	Parameter
Operating System	Windows 10	64-bit
Hardware	CPU	Intel(R) Core(TM) i5-8265U CPU@ 1.60 GHz (8CPUs)~1.8 GHz
	GPU	NVIDIA MX230
	RAM	16 GB
Software Tools	Python3.9	Numpy 1.26.4
		Scikit Learn 1.2.2
		Pandas 2.2.2
		Torch 2.1.0
		Matplotlib 3.8.4

**Table 3:** Model hyperparameter settings.

Parameter	Value
TVC Top-K	5
Pattern capture kernel size	3 × 3
Diffusion coefficient (β)	0.5
Series expansion order (K)	4
Local GCN layers	2
dmodel	512

**Table 4:** Prediction Errors of Indicator $b^{*}$ Based on Neural Network Models.

Metric	Temperature	Tad-Transformer	Transformer	Informer	FEDformer
MAE	−40 °C	0.045506	0.152972	0.297153	0.106034
	−18 ± 0.5 °C	0.043748	0.272312	0.342379	0.219091
	−18 ± 2 °C	0.038482	0.226181	0.296962	0.223873
	−18 ± 4 °C	0.037866	0.213942	0.303487	0.229875
	−18 ± 6 °C	0.039760	0.183316	0.293544	0.202038
	−18 ± 8 °C	0.045113	0.158920	0.336063	0.233296
MSE	−40 °C	0.003814	0.038666	0.121983	0.029316
	−18 ± 0.5 °C	0.002705	0.086485	0.148146	0.075863
	−18 ± 2 °C	0.002784	0.064966	0.119606	0.084158
	−18 ± 4 °C	0.002202	0.054965	0.122463	0.081517
	−18 ± 6 °C	0.002485	0.041926	0.113567	0.084490
	−18 ± 8 °C	0.002640	0.029966	0.141175	0.074794
RMSE	−40 °C	0.061759	0.196637	0.349261	0.171220
	−18 ± 0.5 °C	0.052011	0.294083	0.384897	0.275433
	−18 ± 2 °C	0.052681	0.254872	0.345786	0.289864
	−18 ± 4 °C	0.051012	0.243256	0.344703	0.276405
	−18 ± 6 °C	0.049847	0.204759	0.336997	0.290672
	−18 ± 8 °C	0.051382	0.173107	0.375733	0.273485
MAPE	−40 °C	0.046190	0.210684	0.456922	0.152816
	−18 ± 0.5 °C	0.061062	0.368679	0.477160	0.309085
	−18 ± 2 °C	0.055459	0.310241	0.425573	0.315852
	−18 ± 4 °C	0.051354	0.280914	0.411573	0.312338
	−18 ± 6 °C	0.052910	0.235751	0.392690	0.254138
	−18 ± 8 °C	0.055854	0.192653	0.419178	0.292102
MSPE	−40 °C	0.008537	0.064826	0.277057	0.051459
	−18 ± 0.5 °C	0.004987	0.145611	0.280764	0.146400
	−18 ± 2 °C	0.005270	0.109813	0.239168	0.151651
	−18 ± 4 °C	0.003830	0.087335	0.221012	0.143845
	−18 ± 6 °C	0.004211	0.063884	0.199515	0.123153
	−18 ± 8 °C	0.003936	0.041648	0.217936	0.115491

**Table 5:** Overall performance stability of different models across six temperature conditions (mean ± std over five random seeds).

Metric	Tad-Transformer	Transformer	Informer	FEDformer
MAE	0.0418 ± 0.0032	0.1846 ± 0.0153	0.2023 ± 0.0198	0.3116 ± 0.0251
MSE	0.0028 ± 0.0004	0.0528 ± 0.0087	0.0728 ± 0.0112	0.1290 ± 0.0183
RMSE	0.0520 ± 0.0041	0.2245 ± 0.0192	0.2694 ± 0.0210	0.3578 ± 0.0256
MAPE	0.0542 ± 0.0045	0.2610 ± 0.0221	0.2750 ± 0.0253	0.4370 ± 0.0324
MSPE	0.0052 ± 0.0010	0.0850 ± 0.0142	0.0962 ± 0.0150	0.1180 ± 0.0185

**Table 6:** Silhouette coefficients of different clustering algorithms.

Clustering Algorithm	k = 2	k = 3	k = 4	k = 5	k = 6
K-means	0.4717	0.3393	0.3281	0.3037	0.294
K-means++	0.4812	0.3495	0.3368	0.3320	0.3086
Rule-fused K-means++	0.5094	0.5144	0.4932	0.4648	0.4638

**Table 7:** Cluster centers for quality grades of *L. vannamei*.

Cluster	Quality Grade	TVB-N	$b^{*}$	TBARS	${Hardness}_{adj}$	Sample Number	Proportion
0	Low quality	0.5891	0.6705	0.7846	0.5218	306	44.7%
1	Medium quality	0.5585	0.4766	0.6798	0.4671	264	38.6%
2	High quality	0.5472	0.3827	0.6208	0.4004	114	16.7%

**Table 8:** Evaluation metrics of quality grade prediction models based on rule-fused K-means++.

Model	Low Quality			Medium Quality			High Quality		
	P (%)	R (%)	F1 (%)	P (%)	R (%)	F1 (%)	P (%)	R (%)	F1 (%)
Tad-Transformer	92.43	89.33	90.85	92.45	96.08	94.23	90.53	92.44	91.48
Transformer	89.20	88.11	88.65	90.26	90.85	90.55	89.58	90.72	90.15
FEDformer	87.89	86.28	87.08	86.08	88.89	87.46	88.7	89.08	88.89
Informer	82.58	83.84	83.21	80.95	77.78	79.33	82.01	82.35	82.18

## Data Availability

The original contributions presented in this study are included in the article. Further inquiries can be directed to the corresponding authors.

## Appendix A

This appendix provides the detailed prediction errors for TVB-N, TBARS, and Hardness that are not fully presented in the main text. The results cover five evaluation metrics, MAE, MSE, RMSE, MAPE, and MSPE, across four neural network models, Tad-Transformer, Transformer, Informer, and FEDformer, under different storage temperatures.

**Table A1:** Prediction Errors of Indicator TVB-N Based on Neural Network Models.

Metric	Temperature	Tad-Transformer	Transformer	Informer	FEDformer
MAE	−40 °C	0.093474	0.054948	0.460865	0.217728
	−18 ± 0.5 °C	0.107500	0.051027	0.396731	0.289599
	−18 ± 2 °C	0.090648	0.050464	0.396746	0.297664
	−18 ± 4 °C	0.054481	0.056069	0.369459	0.342088
	−18 ± 6 °C	0.043647	0.057981	0.358380	0.325731
	−18 ± 8 °C	0.058004	0.103367	0.445309	0.298039
MSE	−40 °C	0.011313	0.004403	0.252653	0.085631
	−18 ± 0.5 °C	0.013166	0.004270	0.194879	0.109919
	−18 ± 2 °C	0.009525	0.003991	0.193717	0.120714
	−18 ± 4 °C	0.004199	0.004666	0.168747	0.160277
	−18 ± 6 °C	0.002902	0.005011	0.159991	0.178704
	−18 ± 8 °C	0.005224	0.013814	0.231683	0.124837
RMSE	−40 °C	0.106363	0.066354	0.502646	0.292627
	−18 ± 0.5 °C	0.114743	0.065345	0.441451	0.331540
	−18 ± 2 °C	0.097599	0.063173	0.440133	0.347439
	−18 ± 4 °C	0.064799	0.068308	0.410789	0.400346
	−18 ± 6 °C	0.053869	0.070790	0.399988	0.422734
	−18 ± 8 °C	0.072279	0.117532	0.481335	0.353323
MAPE	−40 °C	0.093860	0.055356	0.464083	0.218522
	−18 ± 0.5 °C	0.111848	0.053174	0.413668	0.301344
	−18 ± 2 °C	0.099213	0.055541	0.434685	0.322094
	−18 ± 4 °C	0.065708	0.066388	0.438482	0.403058
	−18 ± 6 °C	0.052841	0.069230	0.427811	0.376288
	−18 ± 8 °C	0.064919	0.115020	0.501665	0.332985
MSPE	−40 °C	0.011375	0.004478	0.256188	0.086007
	−18 ± 0.5 °C	0.014203	0.004625	0.211952	0.118819
	−18 ± 2 °C	0.011370	0.004852	0.232325	0.139138
	−18 ± 4 °C	0.006140	0.006469	0.236285	0.219921
	−18 ± 6 °C	0.004287	0.007107	0.226554	0.229630
	−18 ± 8 °C	0.006439	0.016821	0.293434	0.153925

**Table A2:** Prediction Errors of Indicator TBARS Based on Neural Network Models.

Metric	Temperature	Tad-Transformer	Transformer	Informer	FEDformer
MAE	−40 °C	0.051017	0.110138	0.448032	0.189066
	−18 ± 0.5 °C	0.053693	0.137023	0.469599	0.209772
	−18 ± 2 °C	0.049988	0.136390	0.462813	0.211794
	−18 ± 4 °C	0.063947	0.125944	0.455014	0.205595
	−18 ± 6 °C	0.077402	0.149915	0.470236	0.229284
	−18 ± 8 °C	0.051959	0.175327	0.443128	0.169541
MSE	−40 °C	0.003310	0.016994	0.232825	0.067564
	−18 ± 0.5 °C	0.003915	0.024158	0.250901	0.082275
	−18 ± 2 °C	0.003488	0.023726	0.243112	0.080207
	−18 ± 4 °C	0.005761	0.019955	0.232410	0.074891
	−18 ± 6 °C	0.007729	0.025912	0.243060	0.082593
	−18 ± 8 °C	0.004474	0.034316	0.222807	0.050511
RMSE	−40 °C	0.057535	0.130363	0.482520	0.259932
	−18 ± 0.5 °C	0.062572	0.155429	0.500900	0.286835
	−18 ± 2 °C	0.059059	0.154033	0.493064	0.283209
	−18 ± 4 °C	0.075902	0.141261	0.482089	0.273663
	−18 ± 6 °C	0.087917	0.160972	0.493012	0.287390
	−18 ± 8 °C	0.066888	0.185245	0.472024	0.224746
MAPE	−40 °C	0.063088	0.130646	0.541333	0.231353
	−18 ± 0.5 °C	0.063953	0.158956	0.551622	0.251703
	−18 ± 2 °C	0.059084	0.157233	0.539381	0.250291
	−18 ± 4 °C	0.072460	0.140558	0.510351	0.233169
	−18 ± 6 °C	0.081429	0.157807	0.494590	0.241634
	−18 ± 8 °C	0.056276	0.189938	0.479688	0.184187
MSPE	−40 °C	0.005178	0.022877	0.331727	0.099230
	−18 ± 0.5 °C	0.005603	0.031482	0.339731	0.120738
	−18 ± 2 °C	0.004883	0.030628	0.324486	0.111525
	−18 ± 4 °C	0.007341	0.024461	0.289107	0.097129
	−18 ± 6 °C	0.008500	0.028619	0.267590	0.091102
	−18 ± 8 °C	0.005195	0.039956	0.258646	0.059184

**Table A3:** Prediction Errors of Indicator Hardness Based on Neural Network Models.

Metric	Temperature	Tad-Transformer	Transformer	Informer	FEDformer
MAE	−40 °C	0.010489	0.057012	0.157282	0.097958
	−18 ± 0.5 °C	0.039264	0.091327	0.114898	0.130531
	−18 ± 2 °C	0.034719	0.092402	0.114648	0.191923
	−18 ± 4 °C	0.039036	0.105599	0.104925	0.300762
	−18 ± 6 °C	0.039546	0.074068	0.144633	0.308360
	−18 ± 8 °C	0.038141	0.117857	0.108856	0.309101
MSE	−40 °C	0.000177	0.004884	0.041382	0.017390
	−18 ± 0.5 °C	0.001750	0.012041	0.028138	0.022858
	−18 ± 2 °C	0.001528	0.012166	0.028336	0.050918
	−18 ± 4 °C	0.001831	0.015557	0.026135	0.117579
	−18 ± 6 °C	0.002271	0.007937	0.041229	0.155893
	−18 ± 8 °C	0.001738	0.018561	0.025273	0.124123
RMSE	−40 °C	0.013304	0.069885	0.203426	0.131872
	−18 ± 0.5 °C	0.041832	0.109731	0.167743	0.151188
	−18 ± 2 °C	0.039090	0.110300	0.168333	0.225650
	−18 ± 4 °C	0.042790	0.124728	0.161663	0.342896
	−18 ± 6 °C	0.047655	0.089090	0.203048	0.394833
	−18 ± 8 °C	0.041688	0.136242	0.158975	0.352311
MAPE	−40 °C	0.051276	0.269183	0.777356	0.453707
	−18 ± 0.5 °C	0.209967	0.480986	0.606497	0.673449
	−18 ± 2 °C	0.246590	0.639948	0.768579	1.368074
	−18 ± 4 °C	0.236821	0.646334	0.621895	1.878624
	−18 ± 6 °C	0.388525	0.709715	1.360604	3.012924
	−18 ± 8 °C	0.260648	0.802137	0.718295	2.122467
MSPE	−40 °C	0.004705	0.109181	1.024747	0.351703
	−18 ± 0.5 °C	0.053443	0.335500	0.779780	0.600542
	−18 ± 2 °C	0.082867	0.599785	1.268161	2.774948
	−18 ± 4 °C	0.069830	0.599338	0.898730	4.963371
	−18 ± 6 °C	0.222335	0.736772	3.687204	15.198584
	−18 ± 8 °C	0.085234	0.879606	1.087254	6.197410
